# Triglyceride to High-Density Lipoprotein Cholesterol (TG/HDL-C) Ratio, a Simple but Effective Indicator in Predicting Type 2 Diabetes Mellitus in Older Adults

**DOI:** 10.3389/fendo.2022.828581

**Published:** 2022-02-24

**Authors:** Hongzhou Liu, Jing Liu, Jixiang Liu, Shuanli Xin, Zhaohui Lyu, Xiaomin Fu

**Affiliations:** ^1^Department of Endocrinology, The First Medical Center, Chinese PLA General Hospital, Beijing, China; ^2^Department of Endocrinology, First Hospital of Handan City, Handan, China; ^3^Clinics of Cadre, Department of Outpatient, The First Medical Center, Chinese PLA General Hospital, Beijing, China; ^4^Department of Cerebral Surgery, First Hospital of Handan City, Handan, China; ^5^Department of Cardiology, First Hospital of Handan City, Handan, China

**Keywords:** triglyceride, high-density lipoprotein cholesterol, type 2 diabetes mellitus, T2DM, older population

## Abstract

**Background:**

A simple and readily available biomarker can provide an effective approach for the surveillance of type 2 diabetes mellitus (T2DM) in the elderly. In this research, we aim to evaluate the role of triglyceride to high-density lipoprotein cholesterol (TG/HDL-C) ratio as an indicator for new-onset T2DM in an elderly Chinese population aged over 75 years.

**Methods:**

This longitudinal retrospective cohort study was conducted using a free database from a health check screening project in China. Participants with baseline TG and HDL measurements were enrolled, and the data of T2DM development were collected. The cumulative incident T2DM rates in different quintile groups of TG/HDL-C ratio (Q1 to Q5) were calculated and plotted. The independent effect of baseline TG/HDL-C ratio on T2DM risk during the follow-up period was tested by the Cox proportional hazard model. Subgroup analysis was also conducted to clarify the role of TG/HDL-C ratio in specific populations.

**Results:**

A total of 231 individuals developed T2DM among 2,571 subjects aged over 75 years during follow-up. Regardless of adjustment for potential confounding variables, elevated TG/HDL-C ratio independently indicated a higher risk of incident T2DM [hazard ratio (HR) = 1.29; 95% confidence interval (CI), 1.14–1.47; *P* < 0.01. As compared with the lowest quintile (Q1), elevated TG/HDL-C ratio quintiles (Q2 to Q5) were associated with larger HR estimates of incident T2DM [HR (95% CI), 1.35 (0.85–2.17), 1.31 (0.83–2.06), 1.85 (1.20–2.85), and 2.10 (1.38–3.20), respectively]. In addition, a non-linear correlation was found between TG/HDL-C ratio and the risk of T2DM, and the slope of the curve decreased after the cutoff point of 2.54. Subgroup analysis revealed a stronger positive correlation among male individuals and those with body mass index <24 kg/m^2^.

**Conclusions:**

Increased TG/HDL-C ratio indicates a greater risk of new-onset T2DM regardless of confounding variables. TG/HDL-C ratio is a simple but effective indicator in predicting T2DM in older adults. More future investigations are warranted to further promote the clinical application of TG/HDL-C ratio.

## Introduction

The incidence of type 2 diabetes mellitus (T2DM) in adults has been steadily increasing worldwide in recent years, especially in the world’s middle-income countries (1). Over the past four decades, the global share of Chinese population with diabetes has increased markedly from 18.9% in 1980 to 24.4% in 2014 (2), imposing a large financial burden on patients and healthcare providers. The population of older T2DM patients is growing faster than any other age group in the world. Older patients with T2DM could have a higher risk of cardio-cerebral vascular events, fundus disease, diabetic nephropathy, and peripheral vascular disorders than those without (3–5). However, the frequency of these complications cannot be reduced effectively by strict control of glucose, since T2DM is usually associated with dyslipidemia, hypertension, and visceral adiposity (6). Previous clinical trials have proven that individualized intervention is effective in reducing or delaying the onset of T2DM in a high-risk population (7). Hence, the recognition and intervention of the population with higher risk of T2DM could provide cost-effective and health benefits. The concealed characteristics of T2DM in the elderly inevitably hinder the diagnosis and treatment for them. More efforts should be dedicated to achieve better risk stratification which is essential for the prevention of elderly T2DM patients. Thus, a novel, accurate, and readily tested predictor is required.

As the typical features of T2DM, β-cell dysfunction and insulin resistance (IR) are not practicable to be tested clinically. Thus, simple biomarkers that are associated with β-cell dysfunction or IR might be helpful for screening individuals at risk for T2DM. Dyslipidemia such as increased triglyceride (TG) or low levels of high-density lipoprotein cholesterol (HDL-C) is a major link in the pathogenesis and etiology of T2DM and prediabetic states (8) and often leads to increased risk of developing atherosclerosis in T2DM patients and experimental models (9, 10). It has been found that hypertriglyceridemia can mediate IR and the subsequent consequence of a vicious circle, in which compensatory hyperinsulinemia and IR can further exacerbate hypertriglyceridemia (11, 12). Currently, there have been a few prospective investigations demonstrating that hypertriglyceridemia is associated with impaired glucose tolerance and fasting glucose (13, 14), as well as the development of T2DM (15, 16). On the other hand, several clinical observations have revealed that HDL-C is inversely correlated with the risk of diabetes (17, 18). More importantly, the ratio of triglyceride to high-density lipoprotein cholesterol (TG/HDL-C) has been recognized as a potential predictive marker of IR, which is a critical trigger for the development of T2DM (19, 20). Therefore, the TG/HDL-C ratio might also be a novel and simple predictor for T2DM, yet very few prospective studies have investigated the correlation between the baseline ratio of TG/HDL-C and the risk of T2DM.

According to current available literature, there have been some conflicting opinions regarding the association of TG/HDL-C ratio with incident T2DM. For instance, Vega et al. suggested that the TG/HDL-C ratio is an effective indicator of T2DM in male patients (21), while a few investigations found that a high TG/HDL-C ratio is related with diabetes only in female patients (22). Some studies have observed a positive connection (21, 23), while one research found a null connection (24). Besides, there exists a significant ethnic disparity in the incidence of T2DM among the populations worldwide. The TG/HDL-C ratio has been found to be associated with IR in non-Hispanic whites (25), but less studied in Chinese population. Furthermore, it is noteworthy that the connection of TG/HDL-C ratio with T2DM might not necessarily be linear. To the best of our knowledge, the linear/non-linear effect of the TG/HDL-C ratio on predicting incident T2DM among the Chinese population and the appropriate cutoff value have rarely been reported.

Although recent investigations have suggested that the TG/HDL-C ratio might be a more effective indicator for IR and T2DM-related complications than solitary TG or HDL-C (26), the correlation between TG/HDL-C ratio and T2DM onset has not been clarified entirely in the elderly population. Moreover, few studies have estimated whether the correlation is modified by gender, age, body mass index (BMI), and blood pressure as well as the function of the liver and kidney. At present, only several prospective observations have investigated this issue, and the extent to which the TG/HDL-C ratio is linearly or non-linearly associated with incident T2DM has been controversial (27–29). In this study, we aim to thoroughly elucidate the relationship between the TG/HDL-C ratio and the risk of T2DM in an older Chinese population aged over 75 years based on a public database and to explore the best cutoff value for TG/HDL-C ratio.

## Methods

### Study Design

The original databases of the population cohort were downloaded from the DATADRYAD website (www.datadryad.org), which allows investigators to freely acquire original data. In line with the Dryad Terms of Service, in this research, we performed the analysis based on the data package sorted by Chen et al. (30). The original cohort was designed based on the medical records of a Chinese population who received a health check from 2010 to 2016. The inclusion and exclusion criteria referred to the study of Chen et al. (30). In brief, this data package included individuals with available information including BMI, gender, and fasting plasma glucose value (FPG). All the participants were followed with visit intervals more than 2 years. Ethics approval was obtained in the previous research by Chen et al. and was no longer needed for the current study. In the original article by Chen et al. (30), the authors declared that they have relinquished copyright and relevant ownership of the database. Therefore, this database was used in this longitudinal analysis without violating the authors’ rights. Since a number of studies have found that the age of 75 years is a significant watershed for the deterioration of many body systems as well as the development of diseases including T2DM, thus we included the participants over 75 years old in our analysis. In the present study, we mainly focused on the role of TG/HDL-C. Considering that increased FPG level indicates the condition of prediabetes or even potential diabetes, we also excluded individuals with abnormally increased baseline FPG, so that the confounding effect of baseline FPG can be avoided. We collected the data of the participants including anthropometric information, sociodemographic parameters, biochemical test, and the onset of T2DM. Then, the association between TG/HDL-C ratio and the risk of T2DM was analyzed based on these data.

### Data Collection and Definitions

In the original database, the baseline examinations were performed by using the standardized spreadsheet to acquire the participant’s general information and by testing the laboratory indices under the fasting status. In our longitudinal analysis, the following parameters were collected and analyzed: age, gender, BMI, TG, HDL-C, blood pressure, alanine aminotransferase, aspartate aminotransferase, blood urea nitrogen, endogenous creatinine clearance rate, total cholesterol, low-density lipoprotein cholesterol, FPG, smoking history, alcohol intake, and follow-up outcome (T2DM).

The endpoint of the study was defined as follows: patients were censored at the time of T2DM diagnosis during the follow-up period or otherwise the last follow-up. According to the report by Chen et al. (30), the diagnosis criteria of incident T2DM was defined as FPG of 7.00 mmol/L at least and/or self-reported T2DM.

### Statistical Analysis

Before the statistical analysis, the missing variables of the individuals in the cohort were first supplemented by using multiple imputation, based on replications and a chained equation approach method in the R MI procedure. The MI (multiple imputation) procedure is an approach to impute missing data with *MICE* package in R software environment. The *MICE* package in R helps to impute missing values with plausible data values which are drawn from a distribution specifically designed for each missing datapoint. Data are expressed as mean ± standard deviation and numbers (percentage) for quantitative variables and qualitative variables, respectively. The one-way ANOVA method was used for evaluating the statistical difference of mean values and chi-square test for percentages.

As for the comparison of the baseline characteristics and the incidence of T2DM, the cohort was assigned to five groups based on the quintile values of TG/HDL-C ratio, namely, Q1, Q2, Q3, Q4, and Q5 groups. The Cox proportional hazard model was used to investigate the independent effect of TG/HDL-C ratio upon the risk of incident T2DM. All of the models were adjusted for none (crude model); gender, age, and BMI (model I); and gender, age, BMI, systolic blood pressure, alanine aminotransferase, and creatinine clearance rate (model II). Afterwards, the impact of TG/HDL-C quintile groups (Q2, Q3, Q4, and Q5 versus Q1, respectively) on T2DM risk was also elucidated. The hazard ratios with 95% confidence intervals and corresponding *P*-values were recorded. The Kaplan–Meier curves were plotted for calculating cumulative T2DM rates, and the log-rank test was used to compare T2DM incidence distributions among TG/HDL-C quintile groups. Next, the non-linear feature of the association between TG/HDL-C ratio and incident T2DM was assessed by the multivariate restricted cubic spline model. We chose six knots at quintiles 0%, 20%, 40%, 60%, 80%, and 100%.

Considering the potential modifications and interactions of gender and BMI in the hazard ratio of TG/HDL-C, the gender-stratified (men or women) and the BMI-stratified (cutoff value, 24 kg/m^2^) multivariate Cox regression proportional hazards models were analyzed. As is known, a higher BMI is associated with greater risk of T2DM. Based on the criteria of weight for adults published by the National Health Commission of the People’s Republic of China, BMI ≥24 kg/m^2^ is considered as overweight. Therefore, we divided body mass index into <24 and ≥24 kg/m^2^ since this study was based on a Chinese population.

SAS version 9.2 (SAS Institute Inc, Cary, NC, USA) and Statistical Packages R (version 4.1.0; The R Foundation, Vienna, Austria) were used for data analysis and plotting. The statistical difference was considered significant when the two-sided *P*-value was <0.05.

## Results

### Baseline Characteristics

Of the 3,756 subjects initially identified, 0 and 1,185 of them were excluded because of lacking TG and HDL-C values, respectively. A total of 2,571 individuals aged over 75 years who underwent physical examinations from 2010 to 2016 were finally identified. As shown in **Table 1**, of these 2,571 participants, 1,634 (63.6%) were men and 937 (36.4%) were women, and they had an average age of 79.68 years (SE, 4.06). The mean TG/HDL ratio was 1.16. Based on the quintile values of TG/HDL-C ratio, the study population was allocated to five groups: Q1 group with mean TG/HDL-C ratio of 0.47 (SE, 0.1; *n* = 514), Q2 group with mean TG/HDL-C ratio of 0.72 (SE, 0.06; *n* = 514), Q3 group with mean TG/HDL-C ratio of 0.96 (SE, 0.08; *n* = 514), Q4 group with mean TG/HDL-C ratio of 1.31 (SE, 0.13; *n* = 514), and Q5 group with mean TG/HDL-C ratio of 2.31 (SE, 0.79; *n* = 515). Participants in the higher TG/HDL-C ratio quintile groups had increased values of systolic blood pressure (*P* < 0.001), BMI (*P* < 0.001), alanine aminotransferase (*P* < 0.001), and triglyceride (*P* < 0.001) and deceased levels of HDL-C (*P* < 0.001).

**Table 1:** Baseline characteristics of the participants.

Variable	All Participants (2,571)	Triglyceride to High-Density Lipoprotein Cholesterol					*P*-value
		Q1 (514)	Q2 (514)	Q3 (514)	Q4 (514)	Q5 (515)	
Age	79.68 (4.06)	80.04 (4.42)	80.12 (4.21)	79.46 (3.92)	79.46 (3.87)	79.34 (3.79)	0.002
tg_hdlc	1.16 (0.74)	0.47 (0.10)	0.72 (0.06)	0.96 (0.08)	1.31 (0.13)	2.31 (0.79)	<0.001
Systolic blood pressure	130.37 (12.18)	128.27 (13.13)	129.38 (12.14)	130.79 (12.28)	131.71 (11.37)	131.68 (11.59)	<0.001
Diastolic blood pressure	76.29 (8.93)	75.11 (8.66)	76.14 (9.01)	75.63 (9.12)	77.44 (8.78)	77.12 (8.86)	<0.001
Body mass index	23.96 (3.18)	22.65 (3.10)	23.38 (3.17)	24.04 (3.15)	24.52 (3.00)	25.23 (2.84)	<0.001
Alanine aminotransferase	17.00 [13.30, 22.00]	16.00 [12.25, 19.60]	16.00 [13.00, 20.30]	16.90 [13.30, 22.48]	17.05 [13.93, 22.35]	18.80 [15.00, 24.75]	<0.001
Aspartate aminotransferase	24.00 [21.00, 28.25]	24.00 [21.00, 28.58]	24.00 [20.90, 28.00]	24.00 [21.10, 28.10]	24.00 [21.00, 28.00]	25.00 [21.90, 29.25]	0.027
Blood urea nitrogen	5.08 (0.92)	5.19 (0.91)	5.10 (0.93)	5.04 (0.90)	5.05 (0.93)	5.04 (0.91)	0.041
Creatinine clearance rate	75.09 (11.89)	75.25 (11.69)	75.73 (11.69)	74.55 (11.86)	74.54 (11.94)	75.38 (12.27)	0.401
Total cholesterol	5.01 (0.73)	4.85 (0.73)	4.99 (0.74)	5.05 (0.73)	5.03 (0.71)	5.14 (0.73)	<0.001
Triglyceride	1.51 (0.83)	0.72 (0.17)	1.06 (0.17)	1.34 (0.22)	1.71 (0.32)	2.74 (0.91)	<0.001
High-density lipoprotein Cholesterol	1.38 (0.24)	1.53 (0.22)	1.46 (0.22)	1.40 (0.21)	1.31 (0.21)	1.20 (0.20)	<0.001
Low-density lipoprotein cholesterol	2.90 (0.54)	2.77 (0.54)	2.91 (0.53)	2.97 (0.55)	2.96 (0.54)	2.90 (0.53)	<0.001
Fasting blood glucose	5.14 (0.47)	5.11 (0.46)	5.12 (0.45)	5.15 (0.47)	5.14 (0.47)	5.20 (0.48)	0.019
Gender							0.004
Men	1,634 (63.6)	356 (69.3)	342 (66.5)	306 (59.5)	315 (61.3)	315 (61.2)	
Women	937 (36.4)	158 (30.7)	172 (33.5)	208 (40.5)	199 (38.7)	200 (38.8)	
Smoking							0.012
None	716 (27.8)	162 (31.5)	134 (26.1)	146 (28.4)	132 (25.7)	142 (27.6)	
Occasionally	119 (4.6)	17 (3.3)	39 (7.6)	17 (3.3)	20 (3.9)	26 (5.0)	
Every day	1,736 (67.5)	335 (65.2)	341 (66.3)	351 (68.3)	362 (70.4)	347 (67.4)	
Drinking							0.381
None	40 (1.6)	6 (1.2)	11 (2.1)	10 (1.9)	7 (1.4)	6 (1.2)	
Occasionally	291 (11.3)	61 (11.9)	70 (13.6)	51 (9.9)	60 (11.7)	49 (9.5)	
Every week	2,240 (87.1)	447 (87.0)	433 (84.2)	453 (88.1)	447 (87.0)	460 (89.3)	
Follow-up year	3.2	3.3	3.2	3.1	3.1	3.1	0.998
Type 2 diabetes mellitus	231 (9.0)	33 (6.4)	37 (7.2)	42 (8.2)	55 (10.7)	64 (12.4)	0.003

### Cumulative Incidence of T2DM

**Figure 1:**
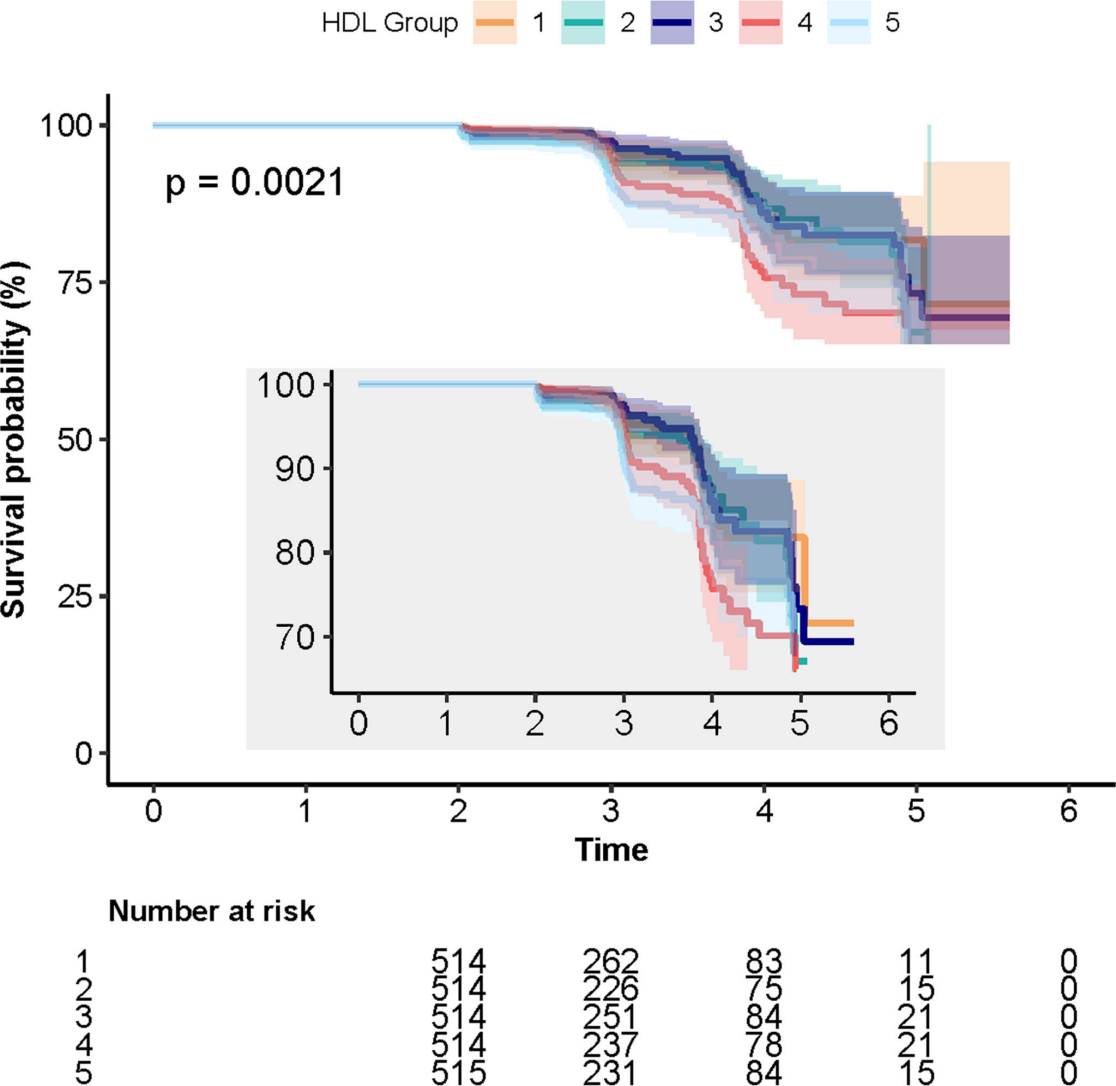
Kaplan–Meier curves depicting the cumulative incident type 2 diabetes mellitus (T2DM) in different quintile groups (Q1 to Q5) of triglyceride to high-density lipoprotein cholesterol ratio. Each color of lines indicates a quintile group. The color range indicates the 95% CI range of cumulative incidence of T2DM at different follow-up times.

A total of 231 cases (9%) of incident T2DM were identified during the follow-up period. The numbers of individuals with T2DM in Q1, Q2, Q3, Q4, and Q5 were 6.4% (*n* = 33), 7.2% (*n* = 37), 8.2% (*n* = 42), 10.7% (*n* = 55), and 12.4% (*n* = 64), respectively. The log-rank test showed that the difference between the five quintile groups was statistically significant (*P* = 0.003; **Table 1**). The 5-year cumulative incidences of T2DM in quintiles 1–5 were 6.4%, 7.2%, 8.2%, 10.7%, and 12.4%, respectively, as shown in **Figure 1**.

### TG/HDL-C Ratio and Incident T2DM

The relationship between TG/HDL-C ratio and T2DM is shown in **Table 2**, calculated by utilizing a Cox proportional hazards regression model. In the crude Cox model, the risk of T2DM significantly increased with elevation of TG/HDL-C ratio (HR = 1.29; 95% CI, 1.14–1.47; *P* < 0.01). The hazard of incident T2DM showed an upward trend in terms of the quintiles of TG/HDL-C ratio (*P* for trend < 0.01). The risk was sustained after being adjusted for gender + age + BMI (multivariable-adjusted model I), gender + age + BMI + systolic blood pressure + alanine aminotransferase + creatinine clearance rate (multivariable-adjusted model II), and gender + age + BMI + FPG (multivariable-adjusted model III). The *P*-values for trend in model I, model II, and model III were 0.01, 0.02, and 0.02, respectively.

**Table 2:** Multivariate analysis for the relationship between TG/HDL-C ratio and incident T2DM.

	Hazard Ratio and 95% CI			
	Crude model	Model I	Model II	Model III
TG/HDL-C	1.29 (1.14, 1.47) <0.01	1.21 (1.06, 1.38) 0.01	1.17 (1.02, 1.34) 0.03	1.18 (1.03, 1.35) 0.02
TG/HDL-C quintiles				
Q1	1	1	1	1
Q2	1.35 (0.85, 2.17) 0.21	1.27 (0.797, 2.03) 0.32	1.24 (0.77, 1.99) 0.37	1.2 (0.75, 1.92) 0.46
Q3	1.31 (0.83, 2.06) 0.25	1.15 (0.73, 1.83) 0.55	1.10 (0.69, 1.75) 0.69	1.06 (0.66, 1.68) 0.82
Q4	1.85 (1.20, 2.85) 0.01	1.61 (1.04, 2.50) 0.03	1.56 (1.01, 2.42) 0.04	1.5 (0.96, 2.32) 0.07
Q5	2.10 (1.38, 3.20) < 0.01	1.72 (1.11, 2.65) 0.01	1.59 (1.02, 2.47) 0.05	1.52 (0.99, 2.35) 0.05
P for trend	<0.01	0.01	0.02	0.02

**Figure 2:**
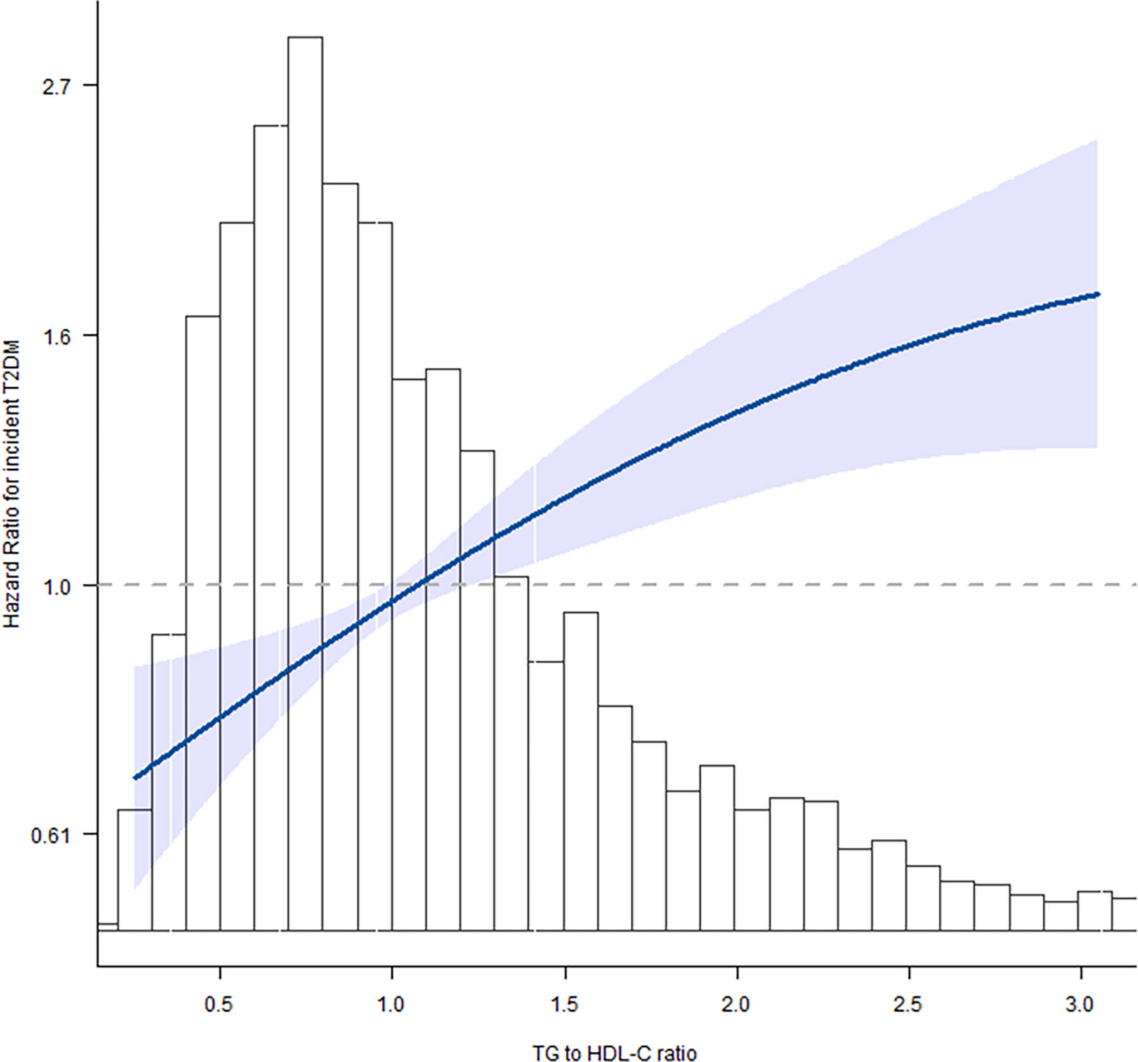
Spline graphical representation of the relationship between triglyceride to high-density lipoprotein cholesterol (TG/HDL-C) ratio and incident T2DM in the multivariable-adjusted model (model II). The restricted cubic splines showed a dose–response relationship between TG/HDL-C ratio variability and incident diabetes. With the increase of TG/HDL-C ratio, the hazard ratio (HR) of incident T2DM gradually increased and showed a curvilinear correlation.

Next, we evaluated the linear/non-linear feature of the association between TG/HDL-C ratio and T2DM by using the multivariate restricted cubic spline analysis. After being adjusted for gender + age + BMI + systolic blood pressure + alanine aminotransferase + creatinine clearance rate, we analyzed the hazard ratios for incident T2DM at six knots of quintiles 0%, 20%, 40%, 60%, 80%, and 100% of TG/HDL-C ratio value. With the increase of TG/HDL-C ratio, the HR of incident T2DM gradually increased and showed a curvilinear correlation (**Figure 2**). The increase of T2DM HR was much lower after the cutoff value of TG/HDL-C ratio = 2.54 as shown in **Table 3**.

**Table 3:** Non-linear effect of TG/HDL-C ratio in incident T2DM.

Outcomes	*P*-value
Linear regression model	<0.01
Non-linear regression model	0.02
Cutoff value of TG/HDL-C	2.54
HR value for TG/HDL-C ratio <2.54	<0.01
HR value for TG/HDL-C ratio ≥2.54	0.54

### Subgroup Analysis

To reduce the potential impact of gender and BMI on the hazard ratio of TG/HDL-C, we further analyzed the association of TG/HDL-C ratio and T2DM in different subgroups (men or women, BMI < 24 or ≥24 kg/m^2^). The following factors were also adjusted: gender, age, BMI, systolic blood pressure, alanine aminotransferase, and creatinine clearance rate. The results are listed in **Table 4**. The results in all the subgroup analysis were similar. The risk of T2DM increased with the quintiles of TG/HDL-C ratio (HR > 1 in all the subgroups).

**Table 4:** Subgroup analysis.

Confounding Factor Category	Serum TG/HDL-C Quintiles					HR for TG/HDL-C as Continuous Variable	*P* for Trend	*P* for Interaction
	Q1	Q2	Q3	Q4	Q5			
Gender								0.62
Men	1	1.21 (0.68, 2.18) 0.52	1.01 (0.56, 1.82) 0.99	1.77 (1.04, 3.00) 0.04	2.09 (1.25, 3.50) 0.01	1.33 (1.12, 1.57) <0.01	0.01	
Women	1	1.57 (0.71, 3.51) 0.27	1.85(0.87, 3.93) 0.11	2.02 (0.95, 4.30) 0.07	2.06 (0.98, 4.32) 0.06	1.24 (1.01, 1.51) 0.04	0.09	
BMI								<0.01
<24	1	1.61(0.84, 3.11) 0.15	1.20 (0.58, 2.47) 0.62	1.85 (0.94, 3.62) 0.07	3.45 (1.80, 6.59) <0.01	1.93 (1.48, 2.52) <0.01	<0.01	
≥24	1	1.01(0.52, 1.98) 0.98	1.08 (0.58, 1.98) 0.82	1.40 (0.78, 2.51) 0.27	1.26 (0.71, 2.23) 0.44	1.09 (0.928, 1.30) 0.32	0.33	

When TG/HDL-C ratio was handled as a continuous variable, the adjusted HRs for T2DM were 1.33 (95% CI, 1.12–1.57; *P* < 0.01), 1.24 (95% CI, 1.01–1.51; *P* = 0.04), 1.93 (95% CI, 1.48–2.52; *P* < 0.01), and 1.09 (95% CI, 0.928–1.30; *P* = 0.32) in the subgroups of men, women, BMI <24 kg/m^2^, and BMI ≥24 kg/m^2^, respectively. The risk of T2DM showed a significantly increased trend from Q1 to Q5 groups in the subgroups of men (*P* for trend = 0.01) and BMI <24 kg/m^2^ (*P* for trend < 0.01) and a non-significant increased trend in the subgroups of women (*P* = 0.09) and BMI ≥24 kg/m^2^ (*P* for trend = 0.33).

## Discussion

So far, this is the first research investigating the relationship between the risk of incident T2DM and TG/HDL-C ratio variability in an older Chinese cohort aged over 75 years. Moreover, restricted cubic splines suggested a non-linear effect of TG/HDL-C ratio in predicting T2DM. These findings were further confirmed in the subgroup analysis, which indicated that this correlation was fairly universal. The effect of dyslipidemia in the development of T2DM has been well studied in previous investigations (9, 10). It has been found that TG/HDL-C ratio is a potential predictive marker for insulin resistance and β-cell dysfunction (25, 26, 31). It has also shown a considerable sensitivity and specificity in predicting incident T2DM (29). However, previous research rarely investigated the role of TG/HDL-C ratio in T2DM development in older individuals aged over 75 years. The current investigation adds to the present knowledge with regard to the correlation between baseline lipid metabolism state and incident T2DM in older Chinese population. After adjusting for conventional risk factors for T2DM including age, BMI, and systolic blood pressure and even further adjusting for gender, alanine aminotransferase, and creatinine clearance rate, the risk of incident T2DM significantly increased with higher baseline TG/HDL-C ratio.

Emerging evidence has suggested that glucose homeostasis is closely associated with lipid metabolism. The development and progression of T2DM can be accelerated by higher TG/HDL-C ratio, TG, and lower HDL-C (29, 32–36). Wagner et al., Heni et al., and Tushuizen et al. revealed that in healthy individuals without diagnosed T2DM, the increased pancreatic lipid content deposition might lead to enhanced dysfunction of β-cells (37–39). Lim et al. also found that the reduction in pancreatic triacylglycerol store might contribute to the normalization of both β-cell function and insulin sensitivity in T2DM (31). Hyperlipidemia facilitates the development of T2DM *via* inducing apoptosis of pancreatic β-cells, the downregulated biosynthesis and secretion of insulin, and abnormality in glucose metabolism. These outcomes are associated with abnormal fatty acid metabolism including lipid deposition, endoplasmic reticulum stress, inflammatory reactions, oxidative stress, and defective insulin signaling, which finally lead to the injury and apoptosis of β-cells (40, 41). Conversely, HDL-C has been indicated to be a potential anti-diabetic factor through ameliorating insulin resistance and β-cell dysfunction (42). On the other hand, in T2DM patients with insulin resistance or hyperinsulinemia, the excessive insulin secretion can regulate the production of lipoprotein lipase and apolipoprotein as well as the function of cholesterol ester transfer protein, thus further promoting the progression of diabetic dyslipidemia (43). Therefore, the vicious circle regarding insulin resistance, β-cell dysfunction, TG, and HDL-C can significantly enhance the risk of T2DM development and progression.

The relationship between TG/HDL-C ratio and T2DM within various TG/HDL-C ratio quintile subgroups might indicate a novel T2DM management and precaution strategy in the elderly. The non-linear effect of TG/HDL-C ratio and its cutoff value associated with T2DM enable it to be used to predict the onset of T2DM as a novel prognostic panel. Previous investigations have shown that abnormalities in a few predictors, including blood pressure, body mass index, triglyceride, high-density lipoprotein cholesterol, low-density lipoprotein cholesterol, and fasting blood glucose, are independently related with not only T2DM but also other disorders such as cardiovascular diseases, cerebral vascular diseases, heart failure, and mortality related with these diseases (44–46). The fluctuation of biomarkers indicates the transformation of physiological modulation and metabolic regulation of the body, as well as the overall impact of life habits and customs, dietary nutrition, and other external interfering factors. These factors are closely associated with the damage and dysregulation of vascular endothelial cell, inflammatory reactions in adipose tissues, insulin resistance, synthesis and secretion of pro-inflammatory cytokines, reactive oxygen species stress, and atherosclerosis (47–50). Elevated levels of these biomarkers might also be indicating the existence of underlying diseases. In clinical practice, the TG/HDL-C ratio could be calculated for each individual at routine health check with the relative indications of low/moderate/high risk of developing T2DM. With the corresponding risk of T2DM, the suggestions such as recheck within 3/6/12 months at an endocrine clinic could also be provided.

The major highlight of the current research is that the investigation of TG/HDL-C ratio and T2DM was conducted in an old population cohort aged over 75 years in China. With the decline of physical function in multiple organs and tissues in the elderly, the characteristic and pathogenesis of T2DM as well as many other diseases might differ from those in the younger population. However, the differences in various age ranges remain largely undetermined. The present study helps to further understand the features of elderly T2DM, as well as the critical role of TG/HDL-C ratio in predicting disease onset. Nevertheless, there were a few limitations. Firstly, this is an observational research which is not applicable for detecting cause–effect correlations. Given that, we performed the subgroup analysis *via* dividing the study sample according to gender and BMI and obtained consistent outcomes. Secondly, we used baseline TG and HDL-C values rather than their variability during follow-up, which might also be a potential limitation. A few investigations suggested that the variability of TG/HDL-C ratio might be of potential predictive value in the onset of T2DM (51). Thus, the role of the combination of baseline and variability of TG/HDL-C ratio in the prognosis of incident T2DM needs to be further investigated in future research. Thirdly, T2DM was diagnosed in the present study without the data of glycosylated hemoglobin (HbA1c) and oral glucose tolerance test (OGTT) results, which might lead to the underestimated incidence of T2DM and missed diagnosis of prediabetes. Nevertheless, a recent report suggested that the FPG-detected prevalence rates of diabetes and prediabetes were 24.3% and 22.8%, respectively. Meanwhile, the OGTT and HbA1c-detected prevalence rates were 6.1% and 4.0%, respectively (52). Therefore, the percentage of missed diagnosis of T2DM individuals or those with prediabetes might be considerably low and not enough to lead to confounding effects in the present study. Lastly, the effect of cardiovascular diseases and concomitant medication was not evaluated due to lack of information in the original database.

## Conclusion

In summary, this study demonstrates that TG/HDL-C ratio is an independent predictive marker for incident T2DM in an older Chinese population aged over 75 years. This finding suggests that regulating the balance of lipid metabolism and stabilization of the TG/HDL-C ratio can be helpful in reducing the risk of developing T2DM. The conclusion should be further verified in larger worldwide epidemiological investigations.

## Data Availability Statement

The original contributions presented in the study are included in the article/supplementary material. Further inquiries can be directed to the corresponding authors.

## Ethics Statement

Ethical review and approval was not required for the study on human participants in accordance with the local legislation and institutional requirements. Written informed consent for participation was not required for this study in accordance with the national legislation and the institutional requirements.

## Author Contributions

ZL and XF contributed to the conception and design of the research. HL, JL, JXL, and SX acquired the data from the database. HL, JL, and SX performed statistical analysis. HL and JL drafted the manuscript. All authors contributed to the article and approved the submitted version.

## Conflict of Interest

The authors declare that the research was conducted in the absence of any commercial or financial relationships that could be construed as a potential conflict of interest.

## Publisher’s Note

All claims expressed in this article are solely those of the authors and do not necessarily represent those of their affiliated organizations, or those of the publisher, the editors and the reviewers. Any product that may be evaluated in this article, or claim that may be made by its manufacturer, is not guaranteed or endorsed by the publisher.
